# Combination effects of ribavirin and interferons on severe fever with thrombocytopenia syndrome virus infection

**DOI:** 10.1186/s12985-015-0412-3

**Published:** 2015-11-02

**Authors:** Masayuki Shimojima, Shuetsu Fukushi, Hideki Tani, Satoshi Taniguchi, Aiko Fukuma, Masayuki Saijo

**Affiliations:** Department of Virology I, Chief, Special Pathogens Laboratory, National Institute of Infectious Diseases, 4-7-1 Gakuen, Musashimurayama, Tokyo 208-0011 Japan

**Keywords:** Combination effect, Interferon, Ribavirin, SFTS, SFTS virus

## Abstract

**Background:**

Severe fever with thrombocytopenia syndrome (SFTS) is an acute infectious disease caused by SFTS virus and characterized by a high case fatality rate. Currently, there is no effective therapy for the disease. While the administration of ribavirin does not improve the case fatality rate or viral load in patient blood, it can inhibit viral infection in vitro.

**Methods:**

Vero cells were pre-treated with interferons (IFNs) α, β, and γ alone and in combination with ribavirin drugs and inoculated with SFTS virus. Three days later, supernatants were harvested and subjected to virus titration. An unpaired t-test was used for statistical analysis of the drugs’ effects.

**Results:**

While the effects of IFNγ at high concentrations were slightly weaker than those of the other IFNs, all IFNs showed dose-dependent inhibitory effects. The combined usage of IFNs with ribavirin at 90 % effective concentrations showed large inhibitory effects, with over a 3 log_10_ reduction in viral titers.

**Conclusions:**

The combined usage of one of type-I/II IFNs with ribavirin drastically reduced SFTS virus infection and therefore may be useful in the treatment of SFTS.

## Background

Severe fever with thrombocytopenia syndrome (SFTS) is a recently-identified tick-born infectious disease characterized by fever, gastrointestinal symptoms, thrombocytopaenia, leukopaenia, and elevated levels of liver enzymes in the peripheral blood [1, 2]. Multiple organ failure and neurologic manifestations are often observed in severe cases [3–5]. The case fatality rate is up to 10 % [6]. The causative agent of the disease is SFTS virus (family *Bunyaviridae*, genus *Phlebovirus*), the discovery of which was reported in 2011 [1, 2]. Currently, no vaccines, therapies, or drugs have proven to be effective against the disease.

It has been reported that SFTS patients treated with ribavirin, plasma exchange, antibiotics, or steroids have recovered [7–11]; however, these reports are deemed to be inconclusive because none of the treatments have been tried with a sufficient number of patients. Liu et al. [12] reported the effects of ribavirin (1-β-D-ribofuranosyl-1,2,4-triazole-3-carboxamide), a guanosine analogue with broad antiviral activities [13], in the treatment of a total of 311 patients, roughly half of whom did not receive ribavirin. In that study, the daily intravenous injection of 500 mg of ribavirin did not significantly affect the case fatality rate or platelet counts and viral load in blood [12], indicating that ribavirin is not effective against SFTS at that dose. However, ribavirin apparently shows an anti-SFTS virus effect in vitro [14]. Thus, a higher dose of ribavirin or a different drug/strategy is likely to be necessary in the treatment of SFTS.

In the present study, we examined the effects of interferons (IFNs) alone and in combination with ribavirin on SFTS virus infection in vitro. The combined usage of IFNs with ribavirin showed large inhibitory effects, suggesting their usability in SFTS treatment.

## Results

### Effects of IFNs on SFTS virus infection

**Fig. 1:**
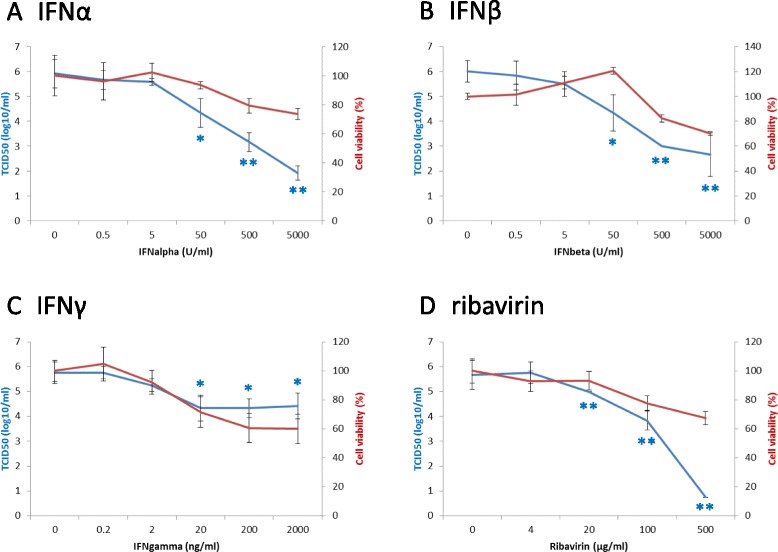
Anti-SFTS virus effects and cytotoxicity of IFNs and ribavirin. To measure effects of IFNs on SFTS virus infection, Vero cells were inoculated with SFTS virus SPL030 strain in the presence of indicated concentrations of IFNα **a**, β **b**, or γ **c**. Titers of supernatants at 3 days post-inoculation are shown in blue. Results are means ± standard deviation obtained from three independent experiments (*n* = 3). To measure cytotoxicity of IFNs, viability of Vero cells cultured in the presence of IFNα **a**, β **b**, or γ **c** was measured by use of WST-1 reagent. Cell viability was calculated as follows: (absorbance of cells in the presence of the drug – absorbance of no cells in the presence of the drug) / (absorbance of cells in the absence of the drug – absorbance of no cells in the absence of the drug) × 100 (%). Experiments were performed in triplicate and means ± standard deviation (*n* = 3) which are shown in red. Effects and cytotoxicity of ribavirin **d** were calculated from the data reported previously [14] to make comparison with those of IFNs easy. Statistical analysis (unpaired t-test in comparison with data from no drug) was performed only for data from anti-SFTS virus effects of IFNs and ribavirin. *,*P* < 0.05; **,*P* < 0.01

Viral titers obtained from Vero cells, which were inoculated in the absence/presence of indicated concentrations of IFNs, are shown in Fig. 1. Although statistically significant inhibitory effects were observed at 50–5,000 U/ml for IFNα and β and 20–2,000 ng/ml for IFNγ, the reduction of viral titers by IFNγ was lower than that by IFNα and β, especially at higher concentrations (Fig. 1a, b, c). Reduction curves were used to calculate 90 % and 99 % effective concentrations (EC_90_ and EC_99_), the drug concentrations at which viral titers were 1 and 2 log-reduced, respectively. The EC_90_ values of IFNα, β, and γ were 29 U/ml, 24 U/ml, and 12 ng/ml, respectively. The EC_99_ values of IFNα, β, and γ were 210 U/ml, 160 U/ml, and >2,000 ng/ml, respectively.

Cytotoxicity was examined by measuring cell viability after cell culture in the presence of drugs without virus inoculation. As shown in Fig. 1, >70 % viability was maintained at all examined concentrations of IFNα and β (Fig. 1a, b), while at 200 and 2,000 ng/ml of IFNγ, cell viability was reduced to 60 % (Fig. 1c). These findings indicate, with the exception of high concentrations of IFNγ (200 and 2,000 ng/ml), the reduced titers of SFTS virus in the presence of IFNs (Fig. 1).

### Combination effects of IFNs and ribavirin

**Fig. 2:**
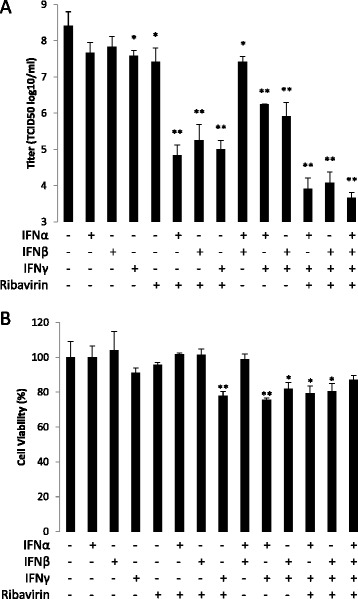
Anti-SFTS virus effects and cytotoxicity of combinations of ribavirin/IFNs. The anti-viral effects **a** and cytotoxicity **b** of ribavirin/IFNs were measured as described in Fig 1. Concentrations of the drugs used were ribavirin 43 μg/ml, IFNα 29 U/ml, IFNβ 24 U/ml, and IFNγ 12 ng/ml, EC_90_ of each drug in the inhibition of SFTS virus infection. Experiments were performed in triplicate and means ± standard deviation are shown (*n* = 3). *,*P* < 0.05; **,*P* < 0.01 (unpaired t-test in comparison with data from no drug)

Using its reduction curve (which has already been reported) [14] (Fig. 1d), the EC_90_ of ribavirin in the inhibition of SFTS virus infection was calculated as 43 μg/ml. The combination effects of each of the IFNs with ribavirin were examined at concentrations of their respective EC_90_ values. At the same time, the single effects of each drug were also examined (Fig. 2a). As expected, when used alone, each drug showed a nearly 1-log reduction of viral titer at its EC_90_ (IFNα, 0.75 log; IFNβ, 0.58 log; IFNγ, 0.83 log; and ribavirin, 1.0 log). Viral titers were greatly reduced by the combined usage of IFNα-ribavirin (3.6 log), IFNβ-ribavirin (3.2 log), and IFNγ-ribavirin (3.4 log). Differences between the effects of the combined usage and those of the single usage were statistically significant (*P* < 0.01, unpaired t-test). The combination effects in the reduction of viral titers were also examined among the three IFNs (α-β, 1.0 log; α-γ, 2.2 log; and β-γ, 2.5 log). The results of triple or quadruple combinations of IFNs and ribavirin, which showed greater inhibitory effects on SFTS virus infection than single- or double-combination-usage of the drugs, are indicated in Fig. 2a.

The examination of cytotoxicity in the combination effect study revealed that >70 % viability was maintained in all combinations (Fig. 2b), while some combinations showed statistically significant difference in comparison with control (no drug) (unpaired t-test).

## Discussion

The combined usage of two or more drugs drastically reduces growth of hepatitis C virus and human immunodeficiency virus (HIV) in vitro and has improved the treatment of both diseases [15, 16]. In the treatment of hepatitis C, the initial drug combination was of broad-spectrum anti-viral agents, IFN and ribavirin. Treatment is now shifting to a combination of IFN and a specific antiviral drug (or drugs), or a combination of specific antiviral drugs [15]. In HIV, the initial efficient combination for treatment was from HIV-specific drugs – in principal, this has not changed, even in recent treatment regimens [16]. While specific antivirals have not yet been developed for the severe acute respiratory syndrome and Middle East respiratory syndrome (MERS) coronaviruses, the combination of IFN and ribavirin has been shown to be effective in both in vitro and in vivo experiments [17–20], and, in the case of MERS, in the treatment of patients [21, 22].

In the present study, the single usage of ribavirin [14] or IFN was shown to significantly reduce the growth of the SFTS virus. Furthermore, it was shown that the combined usage of these drugs at low dosages resulted in large inhibitory effects in vitro. When used singularly, for example, approximately 123 μg/ml of ribavirin [14], 500 U/ml of IFNα, 500 U/ml of IFNβ, or more than 2 μg/ml of IFNγ was necessary to achieve a 3-log reduction of viral growth. In contrast, a combination of 43 μg/ml of ribavirin and either 29 U/ml of IFNα, 24 U/ml of IFNβ, or 12 ng/ml of IFNγ resulted in a >3-log reduction of viral growth. A ribavirin concentration of 43 μg/ml in blood might not be achievable in humans, because the drug reaches a peak serum concentration of 39 μg/ml with doses as high as 2,400 mg injected intravenously [23]. However, in the present study, we used Vero cells, in which higher concentrations of ribavirin are required to inhibit the growth of SFTS virus (and many other viruses) [14], than in other cell types [24, 25], probably due to insufficient phosphorylation of ribavirin to its active triphosphate form in the cell line [26]. Therefore, the administration of high ribavirin doses such as 2,400 mg might show large inhibitory effects against SFTS virus when combined with IFNs. Although the IFNs used in the present study were research-use-only, a concentration of 100-750 U/ml has been observed after the intravenous injection of 3 × 10^7^ U of human-use-approved IFNs [27, 28], which are more than 3 times higher than the IFN concentrations required to reach the EC_90_ of the SFTS virus in vitro. Thus, the combined usage of ribavirin and IFNs at acceptable dosages might result in a large reduction of SFTS virus infection in vivo and could therefore be an effective therapy for the treatment of SFTS patients. Because SFTS virus has been shown to suppress cellular IFN responses [29, 30], the treatment of patients with drugs including IFNs perhaps should be initiated soon after diagnosis of SFTS is done.

In comparison with the combination effects of a single IFN with ribavirin at their respective EC_90_, the combination effects of IFNs without ribavirin on SFTS virus infection, especially the effects of IFNα and β, were small. However, such small combination effects among the IFNs had been expected due to the following reasons: (i) although precise mechanism (s) by which ribavirin affects SFTS virus replication is unclear, ribavirin and IFNs likely work independently of each other [13]; (ii) IFNα and β are type-I IFNs and completely share the receptors that transduce intracellular signals; and (iii) IFNγ is a type-II IFN and partially shares molecules for signal transduction with type-I IFNs [31]. The results, however, strongly suggest that drugs which inhibit SFTS virus proliferation by mechanisms different to those of IFNs/ribavirin could be candidate drugs to be used in combination with IFNs/ribavirin in the treatment of SFTS patients. Further studies may elucidate SFTS virus-specific inhibitors and/or neutralizing antibodies [32] for SFTS therapy.

## Conclusion

The combined usage of one of types I/II IFNs with ribavirin drastically reduced SFTS virus infection and therefore may be useful in the treatment of SFTS.

## Methods

### Cells, virus, and virus titration

Monkey kidney-derived Vero cells (ATCC, CCL-81) were cultured at 37 °C in Dulbecco’s modified Eagle’s medium (DMEM; Sigma) supplemented with 5 % heat-inactivated fetal calf serum (FCS) (Sigma) and antibiotics (Pen Strep, Gibco). Isolation of the Japanese SFTS virus strain SPL030 has been reported [33] and virus titration were performed as described previously [14].

### Effects of drugs on virus infection

Ribavirin, provided by Yamasa-Shouyu Co., Ltd. (Choshi, Japan), was dissolved in phosphate-buffered saline at a concentration of 100 mg/ml. IFNα (universal Type I IFN, PBL InterferonSource), β (human IFN beta 1a, PBL InterferonSource), and γ (rhIFNγ, R & D Systems) were dissolved in DMEM supplemented with 2 % FCS at 500,000 U/ml, 500,000 U/ml, and 200 μg/ml, respectively. For treatment of cells, these drugs were further diluted with cell culture medium at indicated concentration (s), alone or in indicated combinations. Cells were pre-treated with drugs at indicated concentrations for 1 h. One hundred TCID_50_ of SFTS virus was added to the culture without removal of the drugs, and then cultured for 3 days. Culture supernatants were harvested and subjected to virus titration [14].

### Drug cytotoxicity

Cytotoxicity of drugs against Vero cells was measured as described previously [14]. Cells were cultured for 3 days in the presence or absence of the drugs without virus inoculation, and cell viability was measured using the cell proliferation reagent WST-1 (Roche) according to the manufacturer’s protocol. Cell viability was calculated as follows: (absorbance of cells in the presence of the drug - absorbance of no cells in the presence of the drug)/(absorbance of cells in the absence of the drug - absorbance of no cells in the absence of the drug) × 100 (%).

### Statistics

An unpaired t-test was used for statistical analysis of the drugs’ effects.
